# Effects of Abortion Bans on the Physician Workforce

**DOI:** 10.1097/og9.0000000000000206

**Published:** 2026-09-29

**Authors:** Emily Newton-Hoe, Sophia Landay, Subasri Narasimhan, Anitra Beasley, Sara Neill

**Affiliations:** Department of Social and Behavioral Sciences, Harvard T.H. Chan School of Public Health, Boston, Massachusetts; Department of Obstetrics and Gynecology, Beth Israel Deaconess Medical Center, Boston, Massachusetts; UCLA Center on Reproductive Health, Law, and Policy, School of Law, Los Angeles, California; Department of Obstetrics and Gynecology, Baylor College of Medicine, Houston, Texas; and Harvard Medical School, Boston, Massachusetts.

## Abstract

**OBJECTIVE:**

To examine physicians' experiences providing early pregnancy care under abortion bans and perspectives on how these bans affect the reproductive health workforce.

**METHODS:**

From May 2024 to May 2026, we conducted semistructured interviews with obstetrician–gynecologists, family medicine physicians, and emergency medicine physicians practicing in states with total or near-total abortion bans. Participants were recruited with purposive and snowball sampling. Interviews were transcribed verbatim and analyzed with an inductive thematic approach.

**RESULTS:**

Fifty-five physicians from 14 states participated, including 25 obstetrician–gynecologists, 21 emergency medicine physicians, and 9 family medicine physicians. Abortion bans introduced uncertainty that reshaped the boundaries of clinical decision making and narrowed the services physicians felt able to provide, leading to increased referral to specialists for care previously managed by nonspecialists. Participants described tensions between professional obligations and personal well-being, as well as disruptions to core professional values, including commitments to patient autonomy, beneficence, and nonmaleficence. Perceived effects on the training pipeline included loss of expertise, barriers to physician and trainee recruitment, and diminished preparedness for independent practice among trainees in abortion-ban states. Professional burnout emerged as both a consequence of these changes and a factor shaping professional decision making.

**CONCLUSION:**

Physicians across multiple specialties involved in early pregnancy care reported that abortion bans affected scope of practice, physician training, recruitment and retention, and workforce participation. These findings suggest that abortion bans may affect not only abortion provision but also the broader reproductive health care workforce and access to early pregnancy care.

## Abstract 2

Physicians across multiple specialties involved in early pregnancy care reported that abortion bans affected scope of practice, physician training, recruitment and retention, and workforce participation.

Abortion bans implemented after the U.S. Supreme Court's June 2022 *Dobbs v Jackson Women's Health Organization* decision have reshaped reproductive health care delivery in the United States. Research suggests that these bans have contributed to delays in care, increased patient burden, and worsened health outcomes.^1^ These consequences disproportionately affect racialized and economically marginalized populations and may exacerbate long-standing inequities in maternal morbidity and mortality.^2^

Beyond patient care, abortion bans have raised questions about the stability of the reproductive health workforce. Although national data have not demonstrated widespread physician migration after *Dobbs*,^3,4^ studies suggest that abortion bans create enormous workforce strain,^5^ influence local practice patterns,^6^ and disrupt physician training, recruitment, and retention.^7–9^ These issues are particularly salient given projected shortages in both primary care and obstetrics and gynecology nationwide.^10^

Understanding these workforce concerns requires considering the broader reproductive health workforce, which extends beyond obstetrician–gynecologists (ob-gyns) to include family medicine and emergency medicine physicians, who provide essential care in early pregnancy.^11^ Family medicine physicians often provide prenatal and obstetric care, particularly in rural and underserved communities.^12^ Emergency medicine physicians frequently evaluate and manage bleeding in early pregnancy, pregnancy loss, and ectopic pregnancy.^13,14^ These specialties also play critical roles in caring for medically complex and socially vulnerable patient populations.^15^ Although prior qualitative studies have examined how abortion restrictions affect ob-gyns,^8,16,17^ less is known about the experiences of physicians across specialties.

We conducted a qualitative study of physicians whose specialties are core to the reproductive health workforce for early pregnancy care—obstetrics and gynecology, family medicine, and emergency medicine—practicing in states with total or near-total abortion bans. Our objective was to examine physicians' experiences practicing under abortion bans and their perspectives on how these bans affect the reproductive health workforce.

## METHODS

We used purposive and snowball sampling to recruit ob-gyns, family medicine physicians, and emergency medicine physicians who provided early pregnancy care in states with total or near-total abortion bans. Recruitment included email outreach to department chairs and other institutional contacts, who were invited to participate themselves and to distribute study information to physician colleagues. We used neutral outreach language to encourage participation from physicians with a range of perspectives on abortion.

We initially recruited physicians practicing in the 12 U.S. states with total abortion bans as of May 2024 (Fig. 1). Total abortion bans were defined as laws prohibiting induced abortion (with limited exceptions) at all gestational ages. After completion of recruitment in total ban states, we conducted a supplemental purposive sampling phase that included physicians practicing in states with near-total abortion bans, defined as bans at 6 weeks of gestation or at the time of cardiac activity (Fig. 1). This supplemental recruitment was done to explore whether near-total abortion bans had similar effects on the workforce as total bans. No a priori recruitment target was set for either sampling phase; instead, recruitment continued until thematic saturation was reached.^18^

**Fig. 1.**
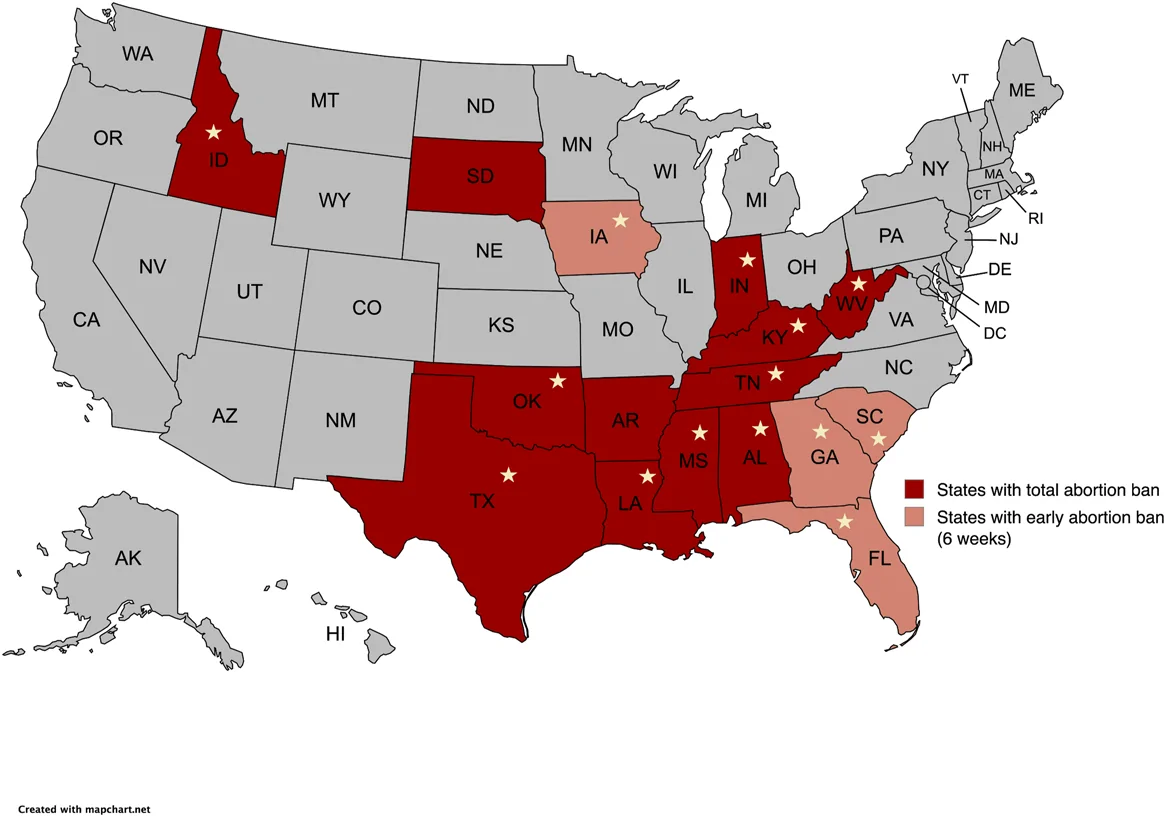
Geographic distribution of study participants by state abortion ban status as of May 2024. *Stars* denote where study participants practice: Alabama, Florida, Idaho, Indiana, Iowa, Georgia, Kentucky, Louisiana, Mississippi, Oklahoma, South Carolina, Tennessee, Texas, and West Virginia. Figure created with MapChart.net 5.4.4.

*Newton-Hoe. Abortion Bans and the Physician Workforce. O&G Open 2026*.

We conducted semistructured interviews through a secure teleconference platform between May 2024 and May 2026. The interview guide included domains related to clinical roles, experiences navigating abortion laws, perceived effects on patient care and training, and decisions about continued practice (Box 1). Interviews were conducted by three team members (two physicians and one public health researcher) with experience in qualitative interviewing and training in qualitative research methods. Interviews lasted approximately 30–45 minutes and were audio-recorded, transcribed verbatim, and deidentified before analysis. Participants provided verbal informed consent and received a $50 gift card as remuneration.

Box 1. **Semistructured Qualitative Interview Guide Domains**

**Domains**

- Clinical training and practice context
- Patient populations and types of reproductive health care provided
- Knowledge and interpretation of relevant abortion and reproductive health laws
- Perceived legal risks and potential penalties for violating state laws
- Changes in clinical practice related to abortion restrictions
- Professional consequences of abortion bans
- Concerns related to personal well-being, safety, or moral distress
- Institutional communication and support related to changes in abortion policy

We analyzed interview transcripts using a thematic approach.^19^ Two team members developed and iteratively refined a codebook through inductive and deductive coding. Each initial transcript was independently coded by two team members in Delve, and discrepancies were resolved through consensus. Once the codebook was consistently applied through consensus coding, subsequent transcripts were coded by a single member of the three-person coding team. Thematic saturation was determined when no new themes emerged.^18^

The multidisciplinary study team included physicians and public health researchers with expertise in reproductive health, whose training and clinical practice span U.S. states with varying protective and restrictive abortion policies. Team members engaged in reflexive discussion throughout data collection and analysis to consider how their professional experiences and positionality might shape their interpretations. The study was approved by the Beth Israel Deaconess Medical Center IRB (2024P000005). The Standards for Reporting Qualitative Research were followed in reporting this article (Appendix 1, available online at https://links.lww.com/AOG/E998).^20^

## RESULTS

Fifty-five physicians from 14 states participated in interviews, including 25 ob-gyns, 21 emergency medicine physicians, and 9 family medicine physicians. Forty-four practiced in states with total abortion bans and 11 in states with near-total abortion bans (Fig. 1). Most participants self-identified as White and female, practiced in the Southern region, and worked in academic or community settings (Table 1). Participants most commonly had 5–9 years of practice experience in their current state (Table 1).

**Table 1.** Characteristics of Study Participants

Characteristics	n (%)
Medical specialty	
Obstetrics and gynecology	25 (45.5)
Emergency medicine	21 (38.1)
Family medicine	9 (16.4)
Practice region	
South	42 (76.4)
Midwest	7 (12.7)
West	6 (10.9)
Abortion-ban status	
Total ban	44 (80.0)
Partial ban	11 (20.0)
Practice setting*	
Academic	37 (67.3)
Community	30 (54.5)
Private practice	7 (12.7)
Years of practice in current state	
Less than 5	18 (32.7)
5–9	20 (36.4)
10–14	8 (14.5)
15–19	3 (5.5)
20 or more	6 (10.9)
Sex	
Female	45 (81.8)
Male	10 (18.2)
Self-described race and ethnicity	
Asian	4 (7.3)
Black	2 (3.6)
Hispanic	1 (1.8)
Mexican	1 (1.8)
Multiracial	4 (7.3)
Prefer not to answer	2 (3.6)
White	40 (72.7)
Unknown	1 (1.8)

Percentages may not add up to 100% because of rounding. Participants self-described their race, ethnicity, and sex using self-identified categories. These variables were collected to describe the sample and to assess generalizability.

* Percentages for practice settings exceed 100% because participants could select more than one setting.

We identified five key themes describing how physicians understood the effects of abortion bans on the reproductive health workforce: narrowing of scope of practice and increasing specialized referrals in response to legal uncertainty; negotiation on decisions to remain in or leave abortion-ban states; disruption of core professional values and ethical principles; effects on the training pipeline; and burnout (Table 2).

**Table 2.** Themes and Illustrative Participant Quotes

Themes	Illustrative Quotes
Narrowing scope of practice and increasing specialized referrals in response to legal uncertainty	“I probably can [provide miscarriage management]. But then there's other laws that say maybe I can't. … I'm very capable of managing this myself, but [I send the patient out of state].” (FM) “I've taken on a lot of the ectopic C-section scar interstitial ectopics, and all these kind of nebulous cases that aren't abortion care. A lot of other providers used to take care of it but now they're like, ‘You take care of it.’ And so I feel like a lot of times, people are very hands off and kind of just shoo things away, and don't want to take on that personal liability. And so that creates a greater burden [on us] as well.” (OB) “[Physicians] in private practice are declining to take care of patients, and they're sending them to academic institutions. To the point where, look, she may have done whatever to find her abortion pills, and she took those medications. But when she presents to the ER, the termination is already done. She's got retained products. It's a whole different diagnosis. But because of the fear that if a provider takes care of her, then they could be involved in this process of her elective termination, they say, go to [redacted institution].” (OB) “When they [patients] come to the ER with a complication, our ob-gyns won't touch them. It's really weird. They will not touch them. They're like, nope, you need to call [the abortion clinic].” (EM)
Negotiating decisions to remain in or leave abortion-ban states	“Part of me feels compelled to stay here because I think there still need to be people on the ground who have some idea about abortion care, even if we can't actually provide the service, so we can consult or refer.” (FM) “Every one of us that leaves just makes it harder for those of us to stay.” (OB) “I am of childbearing age. I have a daughter. I would like to have more children, but I know that if something were to happen, I would have to leave the state to get appropriate medical care, and I don't want to be in that position. I don't want my daughter to be in that position.” (EM) “I recognize that what I love is being able to help my patients, and if I let that [abortion bans] pull me away from my patients, that's not doing anybody any favors.” (EM) “I lost my right to privacy. I've been doxxed. I've had protesters at my house countless times. People sent mailers with my picture and personal information to my ZIP code. I can't make a restaurant reservation in my own name. And I'm sure there's other things that have happened as a consequence [of providing abortion care] that I'm not even aware of. I lost a friend because of that, like a really close friend.” (FM) “I really wanted to stay and be a part of that solution, but ultimately it was just too much to sit and watch people suffer.” (FM)
Disruption of core professional values and ethical principles	“I continue to work at the family medicine residency. Honestly, primarily because I want the residents there to have their training shaped by someone who sees reproductive health topics the way that I do. I don't want them to only hear a conservative anti-choice viewpoint which some of the other faculty members tend to have.” (FM) “There's a point when it becomes too hostile to … practice medicine ethically under the law.” (FM) “I tell [patients] their options. I do. Maybe I'm not supposed to, but you know, you can only do so much for ‘do not harm’ and holding that information [about how to access abortion care], I believe, is doing harm. I'd rather get in trouble than have a patient death on my conscience.” (FM)
Effects on the training pipeline	“Our residents are not allowed to receive abortion training. [Our institution] won't let them travel out of state to get training.” (OB) “[The residents'] elective abortion training has definitely been affected, because prior to post-*Dobbs* changes, we had a pretty good family planning rotation, and they went to a specific place and got all their training. And now, we can't have that training. And so our residents are having to travel, and I think we're on the third or fourth location our residents are going to. … It's been hard that there's not been consistency in one location they can get that training.” (OB) “[Residents] are not going to get the training they really need in terms of pathology … and medication management and what you can offer these patients.” (EM) “[I have] to answer [a question] every interview session about how we are training residents in abortion care, given the limitations that we have in the state.” (OB) “It's challenging to recruit a high quality female applicant here. Because why would they want to come here … especially in these reproductive years … and a point where they're out of school and maybe could be considering having children?” (EM) “We definitely have more people that are like, I don't think I would want to stay [in abortion-restricted state] once I'm done training.” (FM) “[Family medicine trainees] just kind of [send pregnant patients] to the ER, [send] them to OB/GYN without even really trying to figure out what they can do.” (FM) “Residents are going to do training in the community where they might not have OB/GYN. And there definitely has been a sense of like, ‘Oh shoot, what am I allowed to treat? What am I allowed to do? Maybe I'll refer or say go see your OB/GYN doctor tomorrow or send them to the main campus where there is OB/GYN.’ But there's definitely been that expression of fear without having OB/GYN backup.” (EM)
Burnout	“We had some really great younger maternal–fetal medicine [physicians] … and then they all left, like one by one … watching people you respect and seek guidance from just leave because it's unworkable for them… .” (OB) “I think there's unnecessary stress and burden that's placed on me individually because of these changes, because I feel torn from being able to provide the care that our patients need in a timely manner, but also keeping the legal atmosphere in mind when making certain decisions. When I'm on call, it's like, does she really need a repeat hCG or can we just offer her a D&C or offer her mife/miso. So it's just undue or unnecessary stress and burden.” (OB) “The year and a half post-*Dobbs* was horrible. I feel like I didn't realize how bad it was until two years out … what a toll it had taken … just how stressful it was and how depressing it was… . I'm the one turning away all of these patients and giving them other resources. That was really, really exhausting.” (FM)

FM, family medicine; OB, obstetrician–gynecologist; EM, emergency medicine; ER, emergency room; ob-gyn, obstetrician–gynecologist; OB/GYN, obstetrics and gynecology; D&C, dilation and curettage; mife/miso, mifepristone/misoprostol.

Across specialties and abortion-ban contexts, legal uncertainty reshaped the boundaries of clinical practice and contributed to a narrowing of services physicians felt able to provide. Many reported altering their practice to avoid perceived legal risk. This included no longer providing aspects of reproductive health care they considered clinically appropriate and felt competent to provide because of concerns about legal liability. One family medicine physician in a total-ban state with poor maternal health care described: “[Abortion bans are] part of my decision to step away from prenatal care… I don't want to provide good medical care and then someone afterwards [sic] deciding … that was illegal.”

Miscarriage management was an area of particular uncertainty. Several physicians reported referring patients out of state for care they felt “very capable of managing” (family medicine) because they feared potential legal repercussions. These referrals were perceived as contributing to delays in care and fragmented patient management. Family medicine and emergency medicine physicians described deferring care to ob-gyns. Some ob-gyns reported deferring care decisions to maternal–fetal medicine. An ob-gyn in a near-total ban state shared: “I don't necessarily think an MFM [maternal–fetal medicine specialist] needs … to say the person in heart failure shouldn't be pregnant… Any ob-gyn can say that… It's put on MFM because the generalists are uncomfortable with taking that statement on. Not because I don't think they can.”

Legal uncertainty extended to contraceptive care. One family medicine physician in a total-ban state described receiving a referral for a pregnant patient with an intrauterine device after another physician declined to remove it because of concerns that a subsequent pregnancy loss could be interpreted as an abortion. The physician expressed concern that their care could be subject to legal scrutiny: “To an outsider, if they saw … this person went into the office, they got this procedure, [to] remove the IUD [intrauterine device], and then the pregnancy ended. Are they going [to] try and bring me to court over it?”

Professional uncertainty also shaped physicians' decisions about whether to remain in abortion-ban states. Across specialties and abortion-ban contexts, many expressed a strong sense of responsibility to continue providing care in settings with limited reproductive health access. One ob-gyn in a total-ban state explained: “I feel like I'm fighting the fight and trying to at least make things as good as they can be, and make sure that our miscarriage management is still standard of care, and that our patients are getting access to care… If I go, then it's just that much worse here.”

At the same time, participants described emotional strain associated with practicing under abortion bans. Several recounted watching colleagues leave because of frustration, burnout, or concerns about their ability to practice ethically. These departures left some physicians feeling professionally isolated and uncertain about their own future in their state.

Others weighed these professional challenges alongside personal and family considerations when deciding whether to stay. Participants described concerns about their own access to reproductive health care and that of family members. Although most participants had not experienced direct threats to their safety, nearly all expressed concern about antiabortion harassment, sharing stories of colleagues being doxxed online, receiving threatening letters, and encountering protesters outside their homes. One family medicine physician in a total-ban state shared: “There's always that security risk. We've had people get doxxed before and different things where it makes people feel unsafe… It's something I always think about.”

Many participants described abortion bans as disrupting core professional values and ethical principles, particularly beneficence, nonmaleficence, and respect for patient autonomy. Across specialties and abortion-ban contexts, participants emphasized tension between legal constraints and their understanding of their professional obligations as physicians.

Several participants felt unable to provide what they considered standard reproductive health care because of abortion bans. Physicians described situations in which legal considerations overrode what they believed was clinically appropriate, contributing to moral distress and concerns about patient care. An emergency medicine physician in a near-total ban state explained: “I get legitimately frustrated that there's all this legal crap that's getting in the way of me being able to provide the care that I know is the best for pregnant patients.” These experiences were frequently characterized as incompatible with their professional identities and ethical responsibilities. One family medicine physician in a total-ban state shared: “I'm literally being gagged to not be able to provide legal, safe services to people.”

Participants also reported tension between their commitment to physician training and restrictions on reproductive health care provision. Some physicians viewed maintaining exposure to abortion and miscarriage management as an important professional responsibility. At the same time, some expressed distress about their ability to model evidence-based care for trainees while practicing under legal constraints. An emergency medicine physician in a total-ban state explained: “You want to train residents to do the right thing … and I'm not really sure that's an option here anymore.” Participants described how their residency programs were unable to offer abortion training or facilitate out-of-state rotations for trainees. Although some programs incorporated simulation and didactic approaches to mitigate these gaps, participants consistently viewed these efforts as insufficient substitutes for clinical experience. Many expressed concern that trainees were graduating with limited exposure to evidence-based reproductive health care (Table 2).

Recruitment and retention of medical students and residents emerged as major concerns. Participants described how applicants raised questions during interviews about both training opportunities and their own reproductive autonomy. Several participants perceived that trainees increasingly planned to leave abortion-ban states after completing training. Others expressed concern that recruitment difficulties could affect the distribution of the future physician workforce. One ob-gyn in a total-ban state explained: “If we get lower quality applicants, which is what is happening, then those are the people who are going to stay [in our state] and go out into rural areas and care for people.” Concerns about workforce sustainability extended to faculty, with some participants describing “absolute desperation” (emergency medicine) to fill physician positions.

Across specialties and abortion-ban contexts, participants expressed concern about trainee preparedness for independent clinical practice. Some described increased reliance on ob-gyns for reproductive health conditions previously managed within family or emergency medicine. Participants also observed shifts in trainee clinical reasoning, with legal considerations prioritized along with or above medical judgment. One emergency medicine physician in a total-ban state recalled: “Instead of [the resident] telling me her reasoning based on the medical care and the health of the patient, she was citing law…. That was a sad moment for me.”

Burnout both contributed to and compounded concerns. Across specialties and abortion-ban contexts, participants described the mental and physical burden of providing early pregnancy care under restrictions. One ob-gyn in a total-ban state explained how she had taken on leadership and advocacy at her hospital after *Dobbs*, explaining: “I always have to keep my phone on. I always have to be near a computer. Wherever I am in the world, I'm always on standby… I can never really take a break. My brain is always on.” This increased burden often came at the expense of participants' own well-being, with some describing starting antianxiety medications, disrupted sleep, weight gain, and relationship strain.

Participants additionally described the “unnecessary stress and burden” (ob-gyn) involved with providing patient care under abortion bans. A family medicine physician in a near-total ban state described the significant emotional toll of having to turn away patients seeking abortion care, saying: “Every single patient I was counseling [I would say] ‘Legally, we're not allowed to provide your care today, even though it's safe.’ That was draining.” Professional tensions between providing timely care and taking precautionary measures such as performing serial tests to minimize risk further contributed to feelings of burnout.

Participants also described taking on additional responsibilities related to trainee education and supervision. Some reported assuming responsibility for patient care given resident discomfort. Others described spending substantial time educating trainees about state abortion laws, noting that trainees lacked an understanding of legal restrictions in their state. Some participants facilitated out-of-state abortion rotations. The cumulative burden of these additional responsibilities further contributed to burnout.

## DISCUSSION

In this qualitative study of obstetrics and gynecology, family medicine, and emergency medicine physicians, participants described abortion bans as reshaping the reproductive health workforce through a narrowing of services physicians felt able to provide, uncertainty of professional futures and practice locations, and disruptions to training and personal identity. Unlike other prior studies focused primarily on obstetrics and gynecology physicians, our findings suggest that abortion restrictions—including total and near-total bans—influence clinical decision making and workforce dynamics across multiple specialties involved in early pregnancy care. These perceived consequences extended beyond induced abortion to miscarriage management and contraceptive care.

Our findings align with prior research describing moral distress and professional strain among ob-gyns practicing under abortion restrictions.^8,16,17,21,22^ In this study, these findings extend beyond ob-gyns; participants from all three specialties described fear of legal scrutiny and uncertainty regarding the clinical care they could legally provide. Rather than solely affecting care in legally ambiguous situations, this uncertainty contributed to anticipatory compliance. Physicians avoided or deferred aspects of early pregnancy care that they viewed as within their clinical scope of practice because of concerns about future liability. Deferred care resulted in increased referral to more specialized physicians. Family medicine and emergency medicine physicians frequently deferred to ob-gyns, who in turn deferred to maternal–fetal medicine specialists. Increased deferral to ob-gyns or maternal–fetal medicine specialists may disrupt multiple entry points into reproductive health care, particularly in rural and underserved settings where non–obstetrics and gynecology physicians often provide initial evaluation and management of early pregnancy conditions.^12–15,23^ In addition, shifting the management of these patients to maternal–fetal medicine specialists may increase demands on an already limited subspeciality workforce, with potential downstream effects on access to obstetric care and the availability of maternal–fetal medicine services for other high-risk pregnancies.^24^

These findings suggest that abortion bans may undermine efforts to sustain the health care workforce. National workforce initiatives have emphasized supportive work and learning environments, reduction of policy barriers that interfere with clinical care, and improved recruitment and retention.^25^ Physicians in our study identified challenges across each of these domains. Although institutional support and clear clinical guidance may help mitigate some of this strain,^26^ participants' experiences suggest that restrictive policy environments may still affect the long-term capacity of the reproductive health workforce, with some effects potentially taking years to emerge.^5,27^ These concerns are particularly important in the context of projected shortages in primary care and obstetrics and gynecology,^10^ which could worsen if physicians narrow their scope of pregnancy-related care or leave abortion-ban states.

Beyond current clinical practice, concerns about physician training were prominent across specialties and abortion-ban contexts. Consistent with prior research, participants reported reduced opportunities for hands-on reproductive health training and increased reliance on simulation or didactic approaches that they viewed as insufficient substitutes for clinical experience.^28^ Participants described shifts in trainee clinical reasoning, with legal considerations sometimes prioritized alongside or above medical judgment. Perceived changes in trainee recruitment and clinical competence parallel evidence of workforce and training shifts in obstetrics and gynecology after *Dobbs.*^8,9^ Our findings suggest that these effects extend beyond states with total abortion bans and may influence family medicine and emergency medicine trainees. As a result, trainees across a broader range of states and specialties may be less prepared to independently manage early pregnancy care. Because physicians often practice in the states where they train,^29^ these shifts may have longer-term implications for the distribution of the reproductive health workforce and access to early pregnancy care.

This study has several strengths. We included physicians from multiple specialties involved in early pregnancy care and from states representing different geographic regions in the United States. Our participant cohort reflected more than 420 years of experience providing care in their states, and most had grown up or trained in regions where abortion bans are most common (the South and Midwest), giving them deep familiarity with the social and cultural contexts of these states and the ways that abortion bans have affected the physician workforce. The multidisciplinary study team included physicians and researchers with lived and professional experience in both abortion-restrictive and abortion-permissive settings, supporting reflexive interpretation of the data.

A few limitations should be noted. These findings reflect physicians' perspectives and experiences and cannot directly assess quantitative workforce changes. We captured only the perspectives of physicians who remained in states with abortion bans, which may differ from physicians who chose to leave.

In conclusion, participants understood abortion bans as reshaping scope of practice, professional obligations, training environments, and workforce sustainability across multiple specialties involved in reproductive health care. Policies that create legal uncertainty in early pregnancy care may have consequences extending beyond abortion provision to the broader physician workforce responsible for miscarriage management, contraception, and other forms of evidence-based reproductive health care. Future research should examine how abortion bans affect other members of the reproductive health workforce, including nurses and advanced practice clinicians, to better understand the full workforce implications of abortion restrictions.
